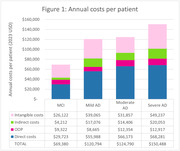# Total Healthcare Costs across the Alzheimer’s Disease Continuum in the United States (US)

**DOI:** 10.1002/alz.086386

**Published:** 2025-01-09

**Authors:** Amir Abbas Tahami Monfared, Noemi Hummel, Emi Naslazi, Artak Khachatryan, Raymond Zhang, Ran Gao, Quanwu Zhang

**Affiliations:** ^1^ Alzheimer’s Disease & Brain Health, Eisai Inc., Nutley, NJ USA; ^2^ McGill University, Montreal, QC Canada; ^3^ Certara GmbH, Loerrach Germany; ^4^ Certara, Oss Netherlands; ^5^ Certara Ltd., Sheffield United Kingdom

## Abstract

**Background:**

This study describes the total healthcare costs integrating direct, indirect, and intangible or emotional cost components across the severity stages of Alzheimer’s disease (AD) in US.

**Method:**

Utilizing Health and Retirement Study (HRS) data (1994‐2018), a bi‐annual US national survey of older adults, we assessed out‐of‐pocket and indirect costs, including unpaid caregiving services, missed workdays, and early retirement. HRS, analyzed with sampling weights, provided a representative US national sample. Mild cognitive impairment (MCI) and AD severity were ascertained using the modified telephone interview of cognitive status (TICS‐M). Besides data from HRS, 100 pairs of patients and their caregivers were referred by physicians and surveyed for quality of life (QoL). Patient and caregiver responses to QoL were converted to utility values. The MCI‐ or AD‐specific health utility value was subtracted from the age‐standardized health utility value from the general US population to derive the estimate of utility reduction due to AD. Intangible costs were calculated using the willingness to pay threshold of $150,000 for one quality‐adjusted life year gained. Direct costs were derived from current literature, comprising patient and caregiver healthcare costs, patient social care costs and nursing home costs. Costs were adjusted to the 2023 US price index.

**Result:**

HRS patient sample (N = 18,786) with MCI (n = 17,885) and AD (n = 901) were aged 67.8±10.7 and 80.9±9.3 years, 55.7% and 63.3% female, and 28.3% and 0.9% employed, respectively. A total of 100 patient‐caregiver pairs with MCI (n = 27) and AD (n = 73) were surveyed, with 21% and 47% of patients > 75 years, 59% and 48% female, and 7.4% and 4.1% employed, respectively. Figure 1 shows mean annual costs per patient across AD severity levels, with direct costs from literature, indirect costs estimated from HRS and intangible costs estimated from the patient‐caregiver survey. Total annual healthcare costs increased from $69,380 for MCI to $150,488 for severe AD.

**Conclusion:**

This study provides a comprehensive view of escalating healthcare costs with worsening AD severity, emphasizing the need for targeted interventions to address the multifaceted economic impact on patients, caregivers, and society.